# First person – Blake Smith

**DOI:** 10.1242/dmm.049122

**Published:** 2021-06-04

## Abstract

First Person is a series of interviews with the first authors of a selection of papers published in Disease Models & Mechanisms, helping early-career researchers promote themselves alongside their papers. Blake Smith is first author on ‘Mouse models for dominant dystrophic epidermolysis bullosa carrying common human point mutations recapitulate the human disease’, published in DMM. Blake is a PhD student in the lab of Ken Pang at Murdoch Children's Research Institute, Parkville, Australia, currently investigating the rare genetic skin disease epidermolysis bullosa utilising a novel mouse model.


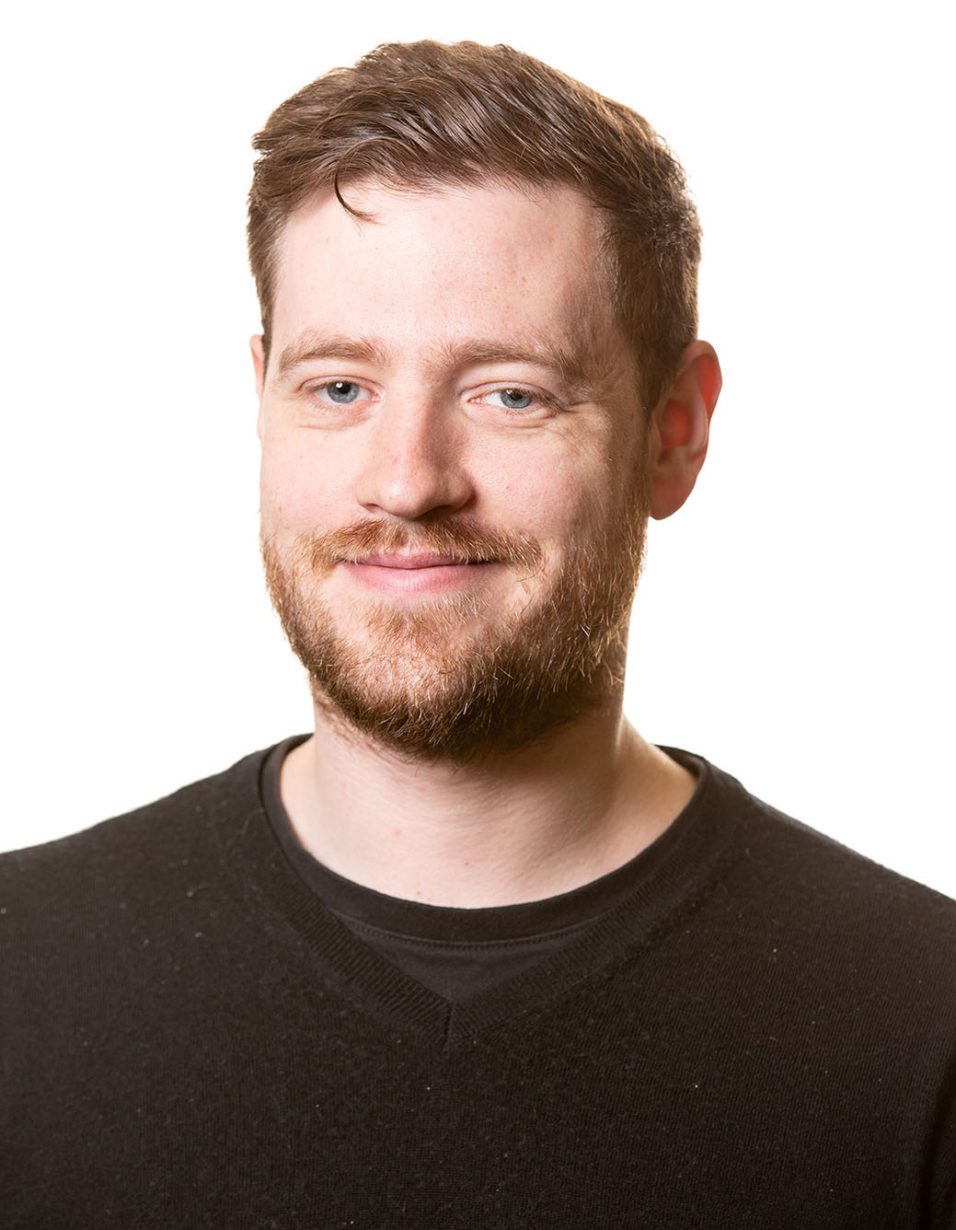


**Blake Smith**

**How would you explain the main findings of your paper to non-scientific family and friends?**

Rare disease research is difficult since the small numbers of patients limit the ability to conduct in-depth, large-scale studies. Animal models of rare diseases can be very useful in such cases, and also help to limit the inherent variability involved in studying patients and their samples. In this paper, we created new mouse models of the rare genetic skin disease dominant dystrophic epidermolysis bullosa (DDEB). DDEB arises when collagen VII, a structural protein that holds the skin layers together, is mutated, and this in turn causes skin fragility and blistering. Importantly, our mouse models recapitulate the main aspects of DDEB that are seen in patients. We see blisters forming on the paws of mice, much like they form on the hands and feet of patients. We also observed a reduction in the amount and stability of collagen VII, which is also seen in patients. These models therefore represent an opportunity for researchers to better understand and develop treatments for DDEB.

“[…] our mouse models recapitulate the main aspects of DDEB that are seen in patients.”

**What are the potential implications of these results for your field of research?**

Having a mouse model for DDEB will allow researchers working in this area to better understand the pathophysiology of this disease and to test novel treatments.

**What are the main advantages and drawbacks of the model system you have used as it relates to the disease you are investigating?**

One of the main advantages of our new mouse models is the relative mildness of their phenotype. In contrast, animal models for other forms of epidermolysis bullosa (EB) are severely affected and frequently result in high infant mortality, which presents ethical and practical barriers to using them within the research setting. A drawback of our mouse models is that – unlike humans – mice have fur, which serves to limit the skin fragility.

**What has surprised you the most while conducting your research?**

**Figure:**
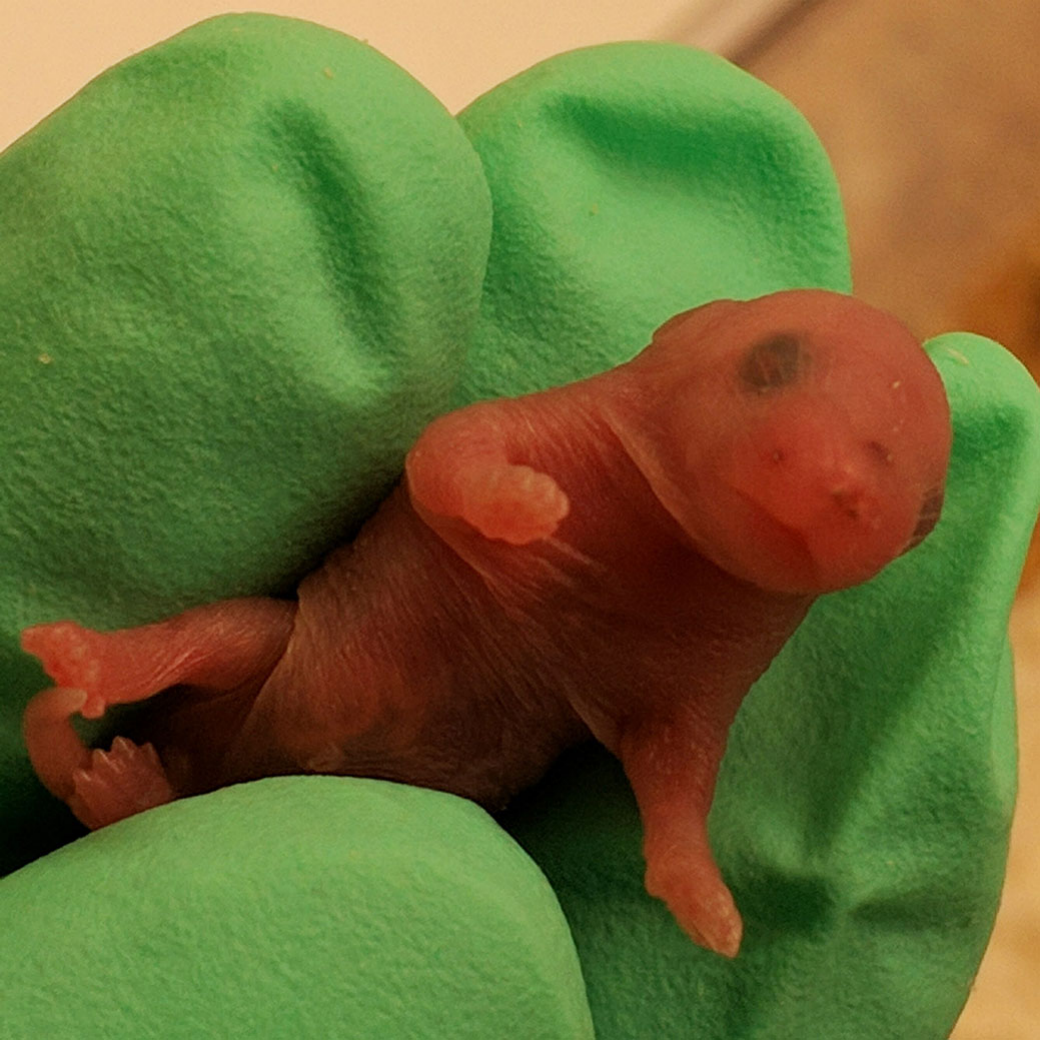
First-generation mouse pup born following the introduction of point mutations via CRISPR-Cas9, with blistering on the top and side of the snout.

There was no guarantee that the common human point mutations we inserted into the mice would lead to a dominantly inherited blistering phenotype in mice, so it was quite a surprise and a relief to see blisters on the paws and mouths of our first generation of newborn mice.

**Describe what you think is the most significant challenge impacting your research at this time and how will this be addressed over the next 10 years?**

Looking ahead, I would like to be involved in developing treatments for EB patients but translating my work from the bench to the bedside. Given the low incidence of EB, patient recruitment for future clinical trials is likely to be challenging. Models such as this will help to address this.

“[…] funding and job security are key issues, and providing greater support for early-career researchers will help to avoid their exodus from a scientific career.”

**What changes do you think could improve the professional lives of early-career scientists?**

I feel that now more than ever that funding and job security are key issues, and providing greater support for early-career researchers will help to avoid their exodus from a scientific career.

**What's next for you?**

I'm fortunate that I will be able to continue working with these mouse models and EB research in an upcoming postdoctoral position.
